# Research progress on FSH-FSHR signaling in the pathogenesis of non-reproductive diseases

**DOI:** 10.3389/fcell.2024.1506450

**Published:** 2024-11-20

**Authors:** Chenhe Li, Yan Ling, Haibin Kuang

**Affiliations:** ^1^ Department of Clinical Medicine, School of Queen Mary, Nanchang University, Nanchang, Jiangxi, China; ^2^ Department of Physiology, School of Basic Medical Sciences, Jiangxi Medical College, Nanchang University, Nanchang, Jiangxi, China; ^3^ Department of Obstetrics and Gynecology, Jiangxi provincial People’s Hospital, Nanchang, Jiangxi, China

**Keywords:** FSH, FSHR, reproduction, menopause, non-reproductive diseases

## Abstract

Follicle-stimulating hormone (FSH), a glycoprotein hormone synthesized and secreted by the anterior pituitary gland, plays a critical role in reproductive development and regulation by binding to FSH receptor (FSHR). Beyond reproductive tissue, FSHRs have been identified in various non-reproductive tissues, indicating broader functions. FSH levels chronically rise during menopause and remain elevated in postmenopausal life. This increase in FSH level has been indicated to be associated with heightened risk of several non-reproductive diseases, including osteoporosis, hypercholesterolemia, type 2 diabetes mellitus, obesity, cardiovascular disease, Alzheimer’s disease, and certain cancers. In this review, we will examine the role of FSH-FSHR signaling in the pathogenesis of these non-reproductive diseases and explore therapeutic strategies targeting FSH-FSHR signaling pathways.

## 1 Introduction

Follicle-stimulating hormone (FSH), a glycoprotein hormone secreted by the anterior pituitary, is primarily recognized for its essential role in reproductive physiology. It can stimulate the development of ovarian follicles in females and spermatogenesis in males, and is regulated by gonadotropin-releasing hormone (GnRH) from the hypothalamus and by steroids feedback of sexual gland, such as estrogen and progesterone (Bernard et al., 2010). Over the past few decades, most research on FSH has been dominated by this classical understanding, which focuses on its crucial roles in reproduction.

However, recent advances in endocrinology have revealed that the influence of FSH extends far beyond its reproductive roles. This is primarily evidenced by the increased identification of FSH extragonadal targets across various tissues and cell types, including bone, pancreatic islets, adipose tissue in different species, such as human and mice (Cheng et al., 2023; Lin and Ge, 2009; Xiong et al., 2023). Growing evidence also suggests that FSH plays a significant role in the pathogenesis of various non-reproductive diseases that have been shown to be more prevalent during menopause, including osteoporosis, type 2 diabetes mellitus, obesity, cardiovascular disease, Alzheimer’s diseases and several cancers (Santoro et al., 2015; Taneja et al., 2019). This paradigm shift in knowledge calls for a more thorough investigation of the non-reproductive role of FSH and its impact on disease development.

Perimenopause, a complex physiological transition typically occurring around the fourth decade of life, is characterized by irregular menstrual cycles and significant hormonal alterations. A decline in estrogen levels concomitant with elevated FSH and luteinizing hormone (LH) concentrations is most notable during this transition (Santoro et al., 2021). While much of the existing literature has focused on the implications of declining estrogen in perimenopausal pathologies, recent studies suggest that elevated FSH may play a more direct and substantial role in the etiology of certain conditions (Gallagher et al., 2010). For instance, the well-documented association between FSH and osteoporosis has catalyzed further exploration into the broader non-reproductive functions of FSH (Cheng et al., 2023; El Khoudary, 2017; Yeh et al., 1996).

FSH mediates its biological effects through interaction with specific G protein-coupled receptors, known as FSHRs. Currently, four isoforms of FSHR (FSHR1, FSHR2, FSHR3, and FSHR4) have been identified across various tissues, each isoform being linked to distinct G protein interactions and subsequent downstream signaling pathways (Simoni et al., 2002). While FSHR1 predominantly governs reproductive functions via cyclic adenosine monophosphate (cAMP)-mediated signaling, other isoforms, such as FSHR3, are implicated in mitogen-activated protein kinase/extracellular signal-regulated kinase (MAPK/ERK) signaling and calcium influx pathways, thereby influencing a wide spectrum of cellular processes beyond reproduction (Li et al., 2007). The presence of FSHRs in non-reproductive tissues, including osteoclasts, adipocytes, vascular endothelial cells, hepatocytes, and neural cells, suggests the potential involvement of FSH in the pathogenesis of various non-reproductive diseases (Casarini and Crepieux, 2019).

This review endeavors to provide a comprehensive update on the current understanding of the non-reproductive functions of FSH and their underlying molecular mechanisms. By examining the influence of FSH on various tissues and its association with disease pathophysiology, this review seeks to elucidate how alterations in FSH levels contribute to increased disease risks. Additionally, this review will explore the potential of FSH-related treatment strategies and discuss future potential directions for research focusing on the non-reproductive roles of FSH.

## 2 FSH and osteoporosis

### 2.1 The roles of FSH in osteoporosis

Osteoporosis is a systemic bone disease characterized by reduced bone mass and microstructural damage to bone tissue, leading to increased bone fragility and susceptibility to fractures (Akhter et al., 2007). In women, the risk of developing osteoporosis increases at the perimenopausal ages, which is accompanied by an increase in FSH levels, supporting the hypothesis that osteoporosis is closely linked to hormonal changes during the menopausal transition (Cannarella et al., 2019; Bergamini et al., 2024). In men with hypogonadism, resulting in excess FSH, observational studies have been conducted. The result also shown that elevated FSH posts a negative effect on male bone mess (Giovanelli et al., 2022). Although previous hypotheses largely agree that the drop in estrogen levels during perimenopause contributes to bone loss, research indicates that an increase in FSH is more closely associated with postmenopausal bone loss (Sun et al., 2006). Early research has shown that bone loss is observed in ovariectomized mice with reduced estrogen level and increased FSH level, but in mice that underwent both ovariectomy and hypophysectomy, the degrade of bone loss is reduced. This result suggests a possible effect of pituitary hormone in the process of bone loss (Yeh et al., 1996). In 2006, Sun et al. (2006) showed that FSHβ or FSHR knockout mice do not develop bone loss, but both develop severe hypogonadism with reduced estrogen levels, further suggesting the effect of FSH on bone resorption.

### 2.2 The underlying mechanisms

Subsequent studies have elucidated the mechanisms by which FSH contributes to bone loss. FSHRs coupled to Giα are present in osteoclasts and their progenitors, but not in osteoblasts (Ghinea et al., 2024). By binding to Giα-coupled FSHR on osteoclasts and osteoclast precursors, FSH activates the phosphorylation of extracellular signal-regulated kinase 1 and 2 (ERK1/2), inhibitor of kappa B alpha (IkBa), and protein kinase B (AKT) (Sun et al., 2006). ERK1/2 and IkBa phosphorylation downstream stimulate osteoclast differentiation. The activated AKT signaling pathway does not appear to have a direct influence on osteoclast formation. However, without AKT signaling, FSH-induced osteoclast formation is reduced (Sun et al., 2006). Overall, the main effect of FSH to induce osteoclastogenesis is exerted downstream via the ERK1/2 and IkBa signaling pathways, while AKT acts as a mediator (Sun et al., 2006). In healthy individuals, moderate FSH levels regulate bone remodeling to maintain stable bone mass. However, in pathological conditions or in periods such as postmenopause, the amount of FSH exceeds the normal value and triggers additional differentiation of osteoclasts, which disrupts the balance of bone remodeling (Gorai et al., 1995; Kawai et al., 2004).

### 2.3 Treatment strategies

Given the uncovered mechanisms of postmenopausal osteoporosis, treatment strategies targeting FSH signaling pathways has been investigated. Initially, GnRH is blocked to downstream suppress FSH level. However, the result shows that the blockade of GnRH signaling not only reduces FSH levels but also leads to hypogonadism and a decrease in LH levels, resulting in serious side effects such as hypogonadism and LH depression (Rendina et al., 2010). To achieve a better therapeutic effect with fewer side effects, further studies are focusing on the development of FSH blocking methods. In 2022, an FSH-blocking antibody, MS-Hu6, was developed and evaluated for safety and efficacy of FSH blocking in mice and monkeys. The results suggest that MS-Hu6 safely and effectively blocks FSH functions without affecting the function of other hormones, such as estrogen and LH. This may be due to the abundance of FSHRs in the ovary, allowing the maintenance of normal FSH functions despite reduced FSH levels. In addition, experiments focused on the therapeutic effect of MS-Hu6 in the treatment of osteoporosis and showed that MS-Hu6 can be distributed into the bone, providing the potential to use it to treat osteoporosis with high FSH levels (Gera et al., 2022). However, these pioneering studies did not establish a way to specifically target the bone tissue, and the use of MS-Hu6 has shown a broad spectrum of effects on adipose tissue, central nervous system and cardiovascular system. Future studies could focus on modifying this antibody through different methods like genetic modification and nanotechnology to specifically target FSHRs on osteoclasts, thereby enabling more precise and specific treatment of osteoporosis.

## 3 FSH and metabolic disorders

### 3.1 The role of FSH in metabolic disorders

Metabolic disorders include a number of diseases that result from disruptions in normal metabolic processes. Metabolic disorders often have a poor prognosis due to the complexity of associated complications, including cardiovascular disease and obesity. Several metabolic disorders have been shown to pose a higher risk in postmenopausal women.

Postmenopausal women often exhibit elevated cholesterol levels. A 2019 study involving 278 pre- and postmenopausal women found significantly higher serum FSH, total cholesterol (TC), and low-density lipoprotein cholesterol (LDL-C) levels in postmenopausal women. To isolate the effect estrogen, further experiments were performed using ovariectomized mice treated with exogenous estrogen to avoid estrogen changes via FSH-estrogen feedback loop. Estrogen levels were carefully matched between the experimental and control groups. The results showed that ovariectomized mice administrated additional exogenous FSH had higher TC and LDL-C levels compared to the control group (Guo et al., 2019). This research demonstrates that FSH can affect cholesterol levels independent of estrogen.

Type 2 diabetes mellitus is another common metabolic disorder that often develops following menopause (LeBlanc et al., 2017; Tatulashvili et al., 2021). Type 2 diabetes mellitus is characterized by peripheral insulin resistance or relative insulin deficiency. Several menopause-related factors, including central obesity, insulin resistance, and decreased lean muscle mass, contribute to the increased risk of type 2 diabetes mellitus (Tatulashvili et al., 2021; Paschou et al., 2019; Vryonidou et al., 2015). Elevated FSH levels have been recognized as a significant risk factor in the pathogenesis of this diseases, possibly due to the role of FSH in promoting fat accumulation, inhibiting insulin secretion, and reducing lean muscle mass (Cheng et al., 2023).

Additionally, the reduction in lean muscle mass associated with elevated FSH levels contributes to development of varying degrees of sarcopenia in menopausal women (Sowers et al., 2007; Douchi et al., 2002). Sarcopenia in the elderly can lead to physical frailty, disability, and a reduced quality of life. Severe sarcopenia is also linked to an increased risk of complications, including cardiovascular disease, diabetes, and obesity (Damluji et al., 2023).

### 3.2 The underlying mechanisms

#### 3.2.1 Hypercholesterolemia

The role of FSH in cholesterol metabolism has been increasingly studied, with recent findings identifying FSHR in the liver (Rossouw et al., 2002). Current evidence suggests that FSH contributes to hypercholesterolemia through two primary pathways. First, FSH binding to hepatocytes is thought to activate sterol regulatory element-binding protein, a transcription factor that subsequently upregulates the expression of HMG-CoA reductase (Guo et al., 2019). HMG-CoA reductase is a key enzyme in hepatic cholesterol biosynthesis and its overexpression is associated with the overproduction of cholesterol (Pertusa et al., 2007). Second, FSH can hinder the clearance of LDL-C from the bloodstream. In ovariectomized mice with elevated FSH levels, reduced expression of LDL receptors on hepatocytes was observed, resulting in decreased LDL-C uptake and an overall increase in serum cholesterol levels (Song et al., 2016).

#### 3.2.2 Type 2 diabetes mellitus

The primary mechanism by which elevated FSH levels may contribute to the development of type 2 diabetes mellitus is the inhibition of insulin secretion from pancreatic islets. FSHRs have been identified on β-cells of human pancreatic islets, which exhibit a biphasic response to FSH. Under normal physiological conditions, FSH binds to FSHRs coupled to the Gαs protein, activating the cAMP/protein kinase A (PKA) and calcium signaling pathways. This pathway enhances glucose-stimulated insulin secretion, helping to maintain normal blood glucose levels. However, when FSH levels are elevated, FSHRs coupled to Gαi are activated, subsequently leading to the inhibition of the cAMP/PKA signaling pathway. This consequently leads to reduced insulin secretion. Thus, prolonged exposure to high FSH levels can lead to insulin deficiency and eventual development of type 2 diabetes mellitus (Cheng et al., 2023).

#### 3.2.3 Sarcopenia

FSHRs have been identified in muscle cells, though the direct effects of FSH on muscle tissue is still not well understood (Chen et al., 2018). Nevertheless, several FSH-induced changes have been implicated in the development of sarcopenia. For example, the reduction in insulin production due to increased FSH levels can impair glucose uptake by muscle cells, disrupting the maintenance of muscle mass and function (Li et al., 2022). Furthermore, the increased bone resorption associated with high FSH levels may contribute to sarcopenia through bone-muscle crosstalk, as bone loss is often associated with reduction in lean muscle mass (Li et al., 2019).

### 3.3 Treatment with metabolic disorders

Efforts to alleviate FSH-induced hypercholesterolemia have included the use of FSH antibodies, although results have been inconsistent. A study investigating the effects of FSH antibody treatment in mice with diet-induced obesity has shown that the treatment successfully reversed triglyceride levels, but it did not significantly alter cholesterol (Liu et al., 2017). In contrast, a 2019 study demonstrated that treatment with FSH antibody could inhibit FSH-induced cholesterol synthesis in ovariectomized mice treated with exogenous estrogen (Guo et al., 2019). The different results of FSH antibodies treatment on cholesterol metabolism highlight the need for further research to explore the relationship between FSH and cholesterol metabolism. In addition to FSH antibodies, reduced lipid and cholesterol levels were also observed in women whose FSH levels decreased by more than 30% following estrogen replacement therapy (Song et al., 2016).

For type 2 diabetes, a lifestyle programme involving 1,007 men with impaired glucose tolerance or type 2 diabetes has shown that testosterone treatment can effectively prevent or revert type 2 diabetes. The group receiving testosterone therapy reports a significantly lower oral glucose tolerance compared to the placebo group. However, 7% of patients in the testosterone-treated group experienced serious adverse events, likely attributable to excess testosterone administration (Wittert et al., 2021). The interaction between FSH and testosterone levels is well established (Oduwole et al., 2021). Therefore, future studies should explore the potential of adjuvant therapies targeting FSH to mitigate the adverse effects associated with excessive exogenous testosterone administration.

Currently, there are no specific treatments directly targeting FSH for type 2 diabetes mellitus and sarcopenia. This may be due to the complex pathogenic mechanisms of these diseases, in which changes in FSH levels play only a minor role. However, treatments such as FSH antibodies, hormone replacement therapy, and FSHR blockade have been successfully applied in related conditions, such as osteoporosis, obesity, and hypercholesterolemia. Thus, targeting FSH in the treatment of type 2 diabetes mellitus and sarcopenia presents a promising area for future research (Bergamini et al., 2024; Rendina et al., 2010; Song et al., 2016).

## 4 FSH and obesity

### 4.1 The role of FSH in obesity

Postmenopausal women often experience weight gain and changes in body composition, which further contribute to the development of obesity. Obesity development in the elderly is multifactorial, while aging is the most predominant factor. However, postmenopausal obesity is characterized by specific features, including increased visceral adiposity, central obesity, and reduced lean muscle mass (Sutton-Tyrrell et al., 2010). And an increase in FSH level has been indicated to be a major risk factor for the progression of postmenopausal obesity in women (Zsakai et al., 2015; Floyd et al., 2016).

FSH is implicated in various metabolic changes contributing to obesity, such as cholesterol accumulation and the progression of type 2 diabetes mellitus. These FSH-induced alterations are particularly problematic for elderly women, who may have reduced physiological resistance to such disorders (Jaff et al., 2014; Crowther and Norris, 2012). Among these changes, fat accumulation remains the most potent driver of obesity progression.

FSH has been shown to promote adipogenesis, with FSH treatment leading to an increase in adipose tissue, particularly in the visceral organs (Liu et al., 2017). This results in central and abdominal obesity as prominent features (Liu et al., 2017). The accumulation of fat in these regions triggers a cascade of physiological changes, including increased body mass index and altered fat distribution, which ultimately constitute the progression of obesity (Davis et al., 2012; Donato et al., 2006). Fat accumulations in visceral areas significantly increase the risk of complications such as coronary artery disease and respiratory disorders (Alberti et al., 2005). Postmenopausal obesity is further characterized by increases in waist circumference and waist-hip ratio, which is due to central obesity (Gavaler and Rosenblum, 2003; Sternfeld et al., 1999).

### 4.2 The underlying mechanisms

The effect of FSH to promote fat accumulation is achieved by targeting adipocytes in different adipose tissues. Among three types of adipose tissues in the human body, FSH mainly affects white adipose tissue and beige adipose tissue, while the effect of FSH on brown adipose tissue is unclear due to a small number of FSHR on adipocytes of brown adipose tissue (Bartke, 2017). In white adipose tissue adipocytes, the activation of FSHRs downstream increases cAMP levels, which downstream activate PKA. PKA phosphorylates a series of transcription factors including peroxisome proliferator-activated receptor gamma (PPARγ), CCAAT/Enhancer binding protein alpha (C/EBPα) and cAMP response element-binding receptors (CREB). These phosphorylated transcription factors then facilitate the expression of lipogenic genes such as lipoprotein lipase (Lpl), fatty acid synthase (Fas) and PPARγ in white adipose tissue, promoting the maturation of preadipocytes into mature adipocytes to perform their fat producing and storage functions (Cui et al., 2012).

**FIGURE 1:**
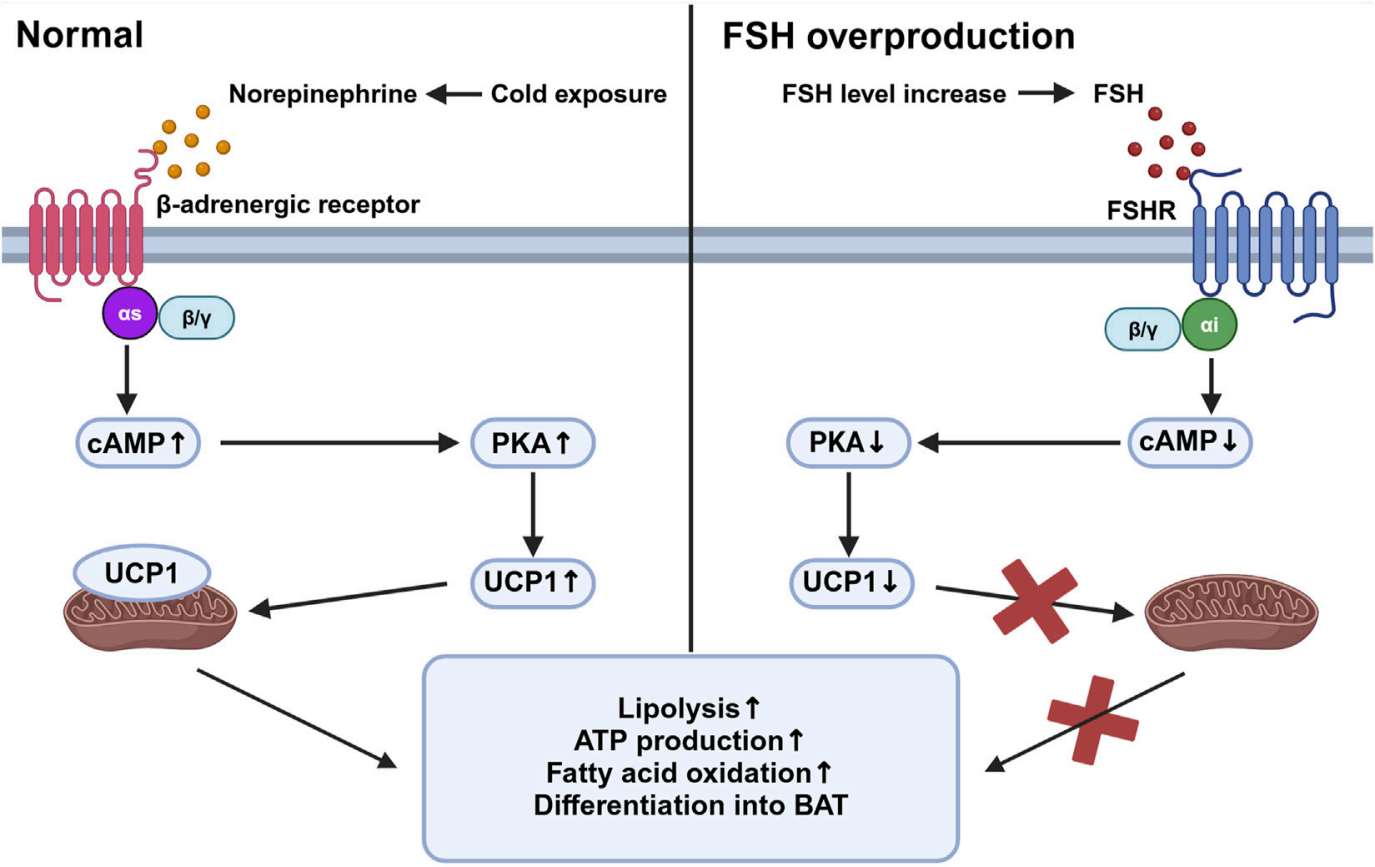
Beige adipose tissue activation under different stimuli. This figure compares the intracellular activities of beige adipose tissue in response to cold exposure (left panel) and elevated follicle-stimulating hormone (FSH) levels (right panel). Under cold exposure, norepinephrine activates β-adrenergic receptors associated with Gαs, stimulating an increased cyclic adenosine monophosphate (cAMP) level which activates protein kinase A (PKA). This pathway upregulates uncoupling protein-1 (UCP1) in mitochondria, promoting lipolysis, ATP production, fatty acid oxidation, and the differentiation of preadipocytes into brown adipose tissue (BAT). Conversely, with elevated FSH levels, FSH binds to follicle-stimulating hormone receptor (FSHR), coupled with Gαi proteins, leading to decreased cAMP levels and reduced PKA activity. This inhibition reduces UCP1 activation, decreasing mitochondrial function and energy expenditure, thereby increasing fat storage and reducing thermogenesis. Created in BioRender. gN, fh. (2024) BioRender.com/d80o007.

Beige adipose tissue is specialized tissue that transforms between white adipose tissue and brown adipose tissue. Studies have shown that FSHR activation blocks the beiging of beige tissue, leading to its fate of white adipose tissue with activation of lipogenesis and inhibition of thermogenesis. In beige tissue, FSHR is coupled to Gαi, which can reduce cAMP and subsequently block the activation of a mitochondrial protein, uncoupling protein-1 (UCP1). UCP1 is a major regulator in beige adipose tissue (Cypess et al., 2009). Normally in the adipocytes of beige adipose tissue, cold exposure triggers the activation of UCP1 mediated by the release of norepinephrine, which facilitates ATP production and oxidation process in abundant mitochondria, allowing the adipose tissue to consume its fat and accelerate metabolic rate. Failure of UCP1 activation in adipose tissue through FSH binding then reduces the consumption of fat tissue and leads to its accumulation and can finally develop into obesity (Marlatt and Ravussin, 2017). The reduction of cAMP also inhibits the process of beige adipose differentiating into brown adipocytes and further increases the expression of lipogenic genes Fas and Lpl (Liu et al., 2017) (Figure 1). In addition, FSH can increase the expression of FSHR in adipocytes and thus amplify its effect (Cui et al., 2012).

### 4.3 Treatment strategies

To overcome the FSH-induced obesity, FSH antibody have been developed and evaluated for their effectiveness and potential as further treatment. Initial experiments were carried out *in vitro* on mice obesity cell line, 3T3-L1 cells. The result shows that FSH antibody treatment to the cell culture can induce the expression of UCP1 as well as other thermogenic genes, including C/EBPα, cell death-inducing DFFA-like effector A (CIDEA), and vascular endothelial growth factor A (VEGFA) (Liu et al., 2017). This finding supports the hypothesis that blocking FSH signaling pathways can stimulate the formation of thermogenic brown adipose tissue, thereby reducing body fat and potentially preventing or curing obesity (Liu et al., 2017).

Subsequent studies utilized the monoclonal FSH antibody, Hf2, to investigate its potential in treating obesity caused by FSH overexpression. Primary research demonstrated that Hf2 can reduce obesity caused by a high fat diet in both female and male mice, indicating the ability of FSH antibody in treating obesity. To further validate the effect of FSH antibody in conditions of excessive FSH production, Hf2 was administrated to ovariectomized mice. The result shows that FSH antibody can reduce the increase of adipose tissue in ovariectomized mice (Greenhill, 2017).

Except for Hf2, recent studies have also demonstrated that another FSHβ antibody, Hu6, also has the effect to reduce adipose tissue in FSH-induced obesity (Bergamini et al., 2024). These findings highlight the potential of using FSH antibodies to prevent and treat obesity driven by FSH overproduction and other factors, such as aging.

## 5 FSH and cardiovascular disease

### 5.1 The roles of FSH in cardiovascular disease

Cardiovascular diseases are currently the leading cause of mortality worldwide, with a significant increased risk observed in postmenopausal women (El Khoudary et al., 2020). Atherosclerosis is the primary underlying mechanism for most cardiovascular disease conditions. The pathogenesis of atherosclerosis involves the accumulation of lipids, inflammatory cells, and fibrous tissue within the arterial walls, which are intricately linked to the endothelium function and lipid metabolism (Yang et al., 2023; Zhao et al., 2019). Previously, the reduction of estrogen during menopause has been considered a key factor in the progression of postmenopausal cardiovascular disease (El Khoudary et al., 2012). However, estrogen replacement therapy has shown limited effectiveness in treating cardiovascular disease, and high-dose estrogen has not been consistently shown to significantly increase the risk of cardiovascular disease, indicating the limited role of estrogen in cardiovascular disease progression (Grodstein et al., 2000; Henderson et al., 1991).

Given this, recent studies have increasingly focused on the role of FSH in cardiovascular disease. Research by Munir et al. (2012) demonstrated a direct correlation between elevated FSH levels and an increased number of aortic plaques. FSH has been linked to conditions that contribute to atherosclerosis, including increased bone resorption and the accumulation of fat and cholesterol (Thomson et al., 2024; Huo et al., 2023). These changes lead to elevated levels of free calcium, fat, and LDL-C within coronary arteries, increasing the risk of carotid intima-media thickness and eventually leading to atherosclerosis (Celestino et al., 2013). Additionally, FSH has been shown to initiate several key processes in the development of atherosclerosis, including adhesion of immune cells and angiogenesis (Moore et al., 2013). In males, recent studies suggest that FSH can exacerbate cardiovascular diseases in the condition of testosterone deficiency, further indicating the FSH effect to promote cardiovascular disease (Duivenvoorden et al., 2024).

### 5.2 The underlying mechanisms

Beyond its impact on fat and calcium metabolism, FSH primarily targets endothelial cells in the progression of cardiovascular disease. Endothelial cell activation is crucial in atherosclerosis development. In most conditions, accumulated lipids and local inflammatory factors trigger the expression of adhesion molecules like intracellular adhesion molecule 1 (ICAM-1) and vascular cell adhesion molecule 1 (VCAM-1) on endothelial cells, recruiting immune cells and forming plaques which further damage the vascular wall and block the lumen (Moore et al., 2013). Elevated FSH level activates endothelial cells through an alternative pathway mediated by FSHR. FSHR in endothelial cells are coupled with Gαs proteins, whose activation downstream increases cAMP concentrations and activate PKA. PKA upregulates VCAM-1 expression and initiate atherosclerosis (Li et al., 2017).

FSH also facilitates angiogenesis, a critical step in atherosclerosis. In most conditions of cardiovascular disease, angiogenesis is driven by VEGF (Shaw et al., 2024). However, studies have indicated that FSH can induce angiogenesis without VEGF. FSH perform their angiogenic function also through the FSHR on endothelial cells, but through the phosphoinositide 3-kinase (PI3K)/AKT pathway. The activation of PI3K/AKT pathway in endothelial cells will mimic the function of VEGF and further trigger angiogenesis (Stilley et al., 2014).

### 5.3 Treatment strategies

Currently, there is no specific treatment for cardiovascular disease targeting FSH or FSHR. FSH antibody or replacement therapies have not significantly reduced atherosclerosis or other conditions of cardiovascular disease. The complexity of cardiovascular disease, involving multiple risk factors such as lifestyle, genetics, and environment, may limit the efficacy of targeting a single risk factor like FSH. However, by targeting FSH signaling, treatment strategies aimed to alleviate symptoms, prevent severe complications, and ensure better prognosis for cardiovascular diseases still hold the potential for future study. For example, we have previously mentioned that FSH-targeting therapies can reduce bone resorption, fat and cholesterol accumulation, which are risk factors that contribute to cardiovascular diseases.

## 6 FSH and Alzheimer’s disease

### 6.1 The roles of FSH in Alzheimer’s diseases

Observational studies utilizing multimodal brain imaging have revealed an increase in amyloid-β (Aβ) deposition in peri-menopausal women (Mosconi et al., 2017). This finding suggests that changes during menopausal transition may contribute to Alzheimer’s disease development. Initial research focused on the role of declining estrogen levels in Alzheimer’s disease progression, but the outcomes of estrogen-replacement therapy in postmenopausal women have been inconsistent, showing effects ranging from exacerbation to improvement of Alzheimer’s disease symptoms (Matyi et al., 2019; Mulnard et al., 2000; Shumaker et al., 2004). In contrast, elevated FSH levels have been shown to correlate with Alzheimer’s disease progression, implicating the elevated FSH as a potential contributor to Alzheimer’s disease pathogenesis (Short et al., 2001).

To specifically investigate the effects of FSH, Xiong et al. (2023) developed an Alzheimer’s disease mouse model through ovariectomy, which disrupts sex hormone levels. Estrogen replacement was employed to maintain normal estrogen levels. This study found an increase in Alzheimer’s disease related markers in the brain, including Aβ and hyperphosphorylated Tau368, indicating that FSH may promote Alzheimer’s disease independently of estrogen. Additionally, treatment with FSH antibodies led to a reduction in these pathological markers, further supporting the notion that FSH plays a role in Alzheimer’s disease development.

These findings establish a relationship between elevated FSH levels and the progression of Alzheimer’s disease, evidenced by increased Alzheimer’s disease hallmarks. More recent studies have demonstrated that FSH directly affects neurons (Short et al., 2001; Xiong et al., 2022; Bowen et al., 2000). Immunofluorescence studies have shown that FSH can increase the levels of Aβ and Tau368 in neurons, which are characteristic of the neurodegenerative process in Alzheimer’s disease (Xiong et al., 2022). Furthermore, FSH administration has been associated with plaque accumulation in the hippocampus and cortex, regions critical for memory and cognition, which led to increased neuronal apoptosis in these areas and increased risk of Alzheimer’s disease (Xiong et al., 2022).

### 6.2 The underlying mechanisms

**FIGURE 2:**
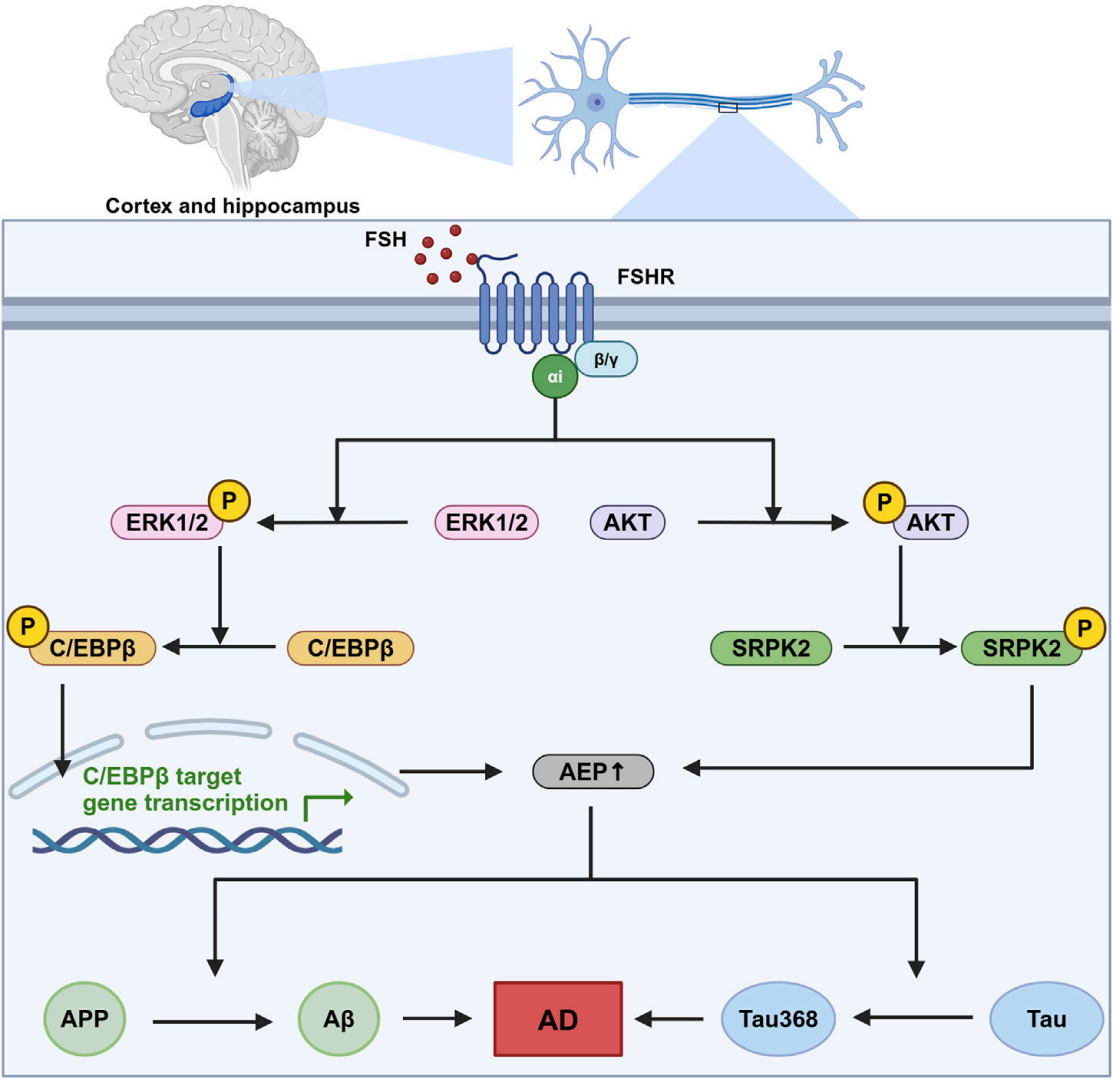
The role of FSH-FSHR signaling in Alzheimer’s disease pathogenesis. This figure illustrates the signaling pathways activated by FSH in neurons located within the cortex and hippocampus. This sequence of molecular events contributes to the neurodegenerative processes that are indicated to cause Alzheimer’s disease (AD), highlighting a novel mechanism by which follicle-stimulating hormone (FSH) could influence AD pathology independent of its canonical reproductive roles. In this pathway, FSH activates follicle-stimulating hormone receptor (FSHR) coupled with Gαi, which downstream phosphorylate CCAAT/Enhancer binding protein alpha (C/EBPβ) and stress-activated protein kinase (SRPK2). These two molecules subsequently enhance the expression and effect of asparagine endopeptidase (AEP), leading to an increased cleavage of amyloid precursor protein (APP) and Tau protein. This pathway ends with the formation of neurodegenerative hallmarks amyloid-β (Aβ) and Tau368. Created in BioRender. gN, fh. (2024) BioRender.com/s03s437.

FSHRs are identified in the central nervous system, particularly in neurons within the cortex and hippocampus (Xiong et al., 2022). In neurons, FSHRs are coupled with the Gαi protein, which downstream induces the phosphorylation of AKT, ERK1/2, and SRPK2. The phosphorylation of these molecules leads to the upregulation of the C/EBPβ/asparagine endopeptidase (AEP) pathway, promoting the accumulation of Aβ and Tau368 (Xiong et al., 2023). C/EBPβ is a transcription factor involved in several biological processes, including energy metabolism, cell proliferation, differentiation, and inflammation (Greenbaum et al., 1998; Poli, 1998; Straccia et al., 2011). In Alzheimer’s disease, C/EBPβ has been associated with neurotoxicity and can be induced by pro-inflammatory factors, contributing to the pathological processes in Alzheimer’s disease. Upregulated C/EBPβ expression subsequently enhances the transcription of δ-secretase, also known as AEP. AEP plays a critical role in cleaving amyloid precursor protein (APP) and Tau, thereby facilitating the accumulation of Aβ and hyperphosphorylated Tau368 (Wang et al., 2018). This sequence of events is a significant contributor to the development and progression of Alzheimer’s disease (Figure 2).

### 6.3 Treatment strategies

Studies have investigated the potential of targeting FSH signaling in the treatment of Alzheimer’s disease. Notably, the knockdown of neuronal FSHR has been shown to inhibit Alzheimer’s disease pathogenesis, with a significant reduction in the production of Aβ and Tau368 (Xiong et al., 2022). This pioneering research highlights the potential therapeutic strategy of blocking FSH action in neurons to mitigate Alzheimer’s disease progression.

Building on this, a recent study employed an FSHβ antibody to treat Alzheimer’s disease mouse models, corroborating earlier findings by demonstrating a reduction in both Aβ levels and AEP activity. The results suggest that inhibiting FSH could have a neuroprotective effect. Interestingly, the study utilized intraperitoneal administration of the FSH antibody, yielding promising results in preventing Alzheimer’s disease pathology. This finding implies that peripheral blockade of FSH might be sufficient to prevent its pathological effects in the central nervous system (Xiong et al., 2023). Although FSH antibodies have shown significant effects in treating FSH-induced neural impairments, future research is in need to explore other treatment strategies such as FSHR blockade specifically in neurons for better treatment effect targeting central nervous system.

## 7 FSH and cancer

### 7.1 The roles of FSH in cancer

FSH has been demonstrated to take part in the pathogenesis of several cancers, including ovarian and breast cancers in females and prostate cancer in males.

#### 7.1.1 Ovarian cancer

Ovarian cancer has the highest mortality rate among all gynecological cancers and ranks fifth in cancer-related mortality in women. A study involving 1,411 women with epithelial ovarian cancer has shown that the incidence of ovarian cancer increases with elevated FSH levels, particularly post-menopause (Schildkraut et al., 2001). Another study observed increased FSH levels in the peritoneal fluid of ovarian cancer patients, further suggesting a link between high FSH levels and ovarian cancer pathogenesis (Halperin et al., 1999). Moreover, studies have indicated that FSH exerts similar effects on ovarian cancer cells as on follicular development, promoting key cancerous processes such as proliferation, migration, invasion, and angiogenesis (Li et al., 2007; Stilley et al., 2014; Liu et al., 2015).

#### 7.1.2 Breast cancer

Breast cancer is the most prevalent cancer type among women globally. Estrogen is the primary hormone implicated in the development of breast cancer. Under normal physiological conditions, estrogen, predominantly produced by the ovaries, plays a crucial role in the development and maintenance of breast tissue. However, in the context of malignancy, estrogen signaling can promote the growth and survival of cancerous cells (Blander, 2006). Beyond estrogen, studies have identified FSHRs on various breast cancer cell lines, such as SK-BR-3, MDA-MB-231, MCF-7, and T-47D. *In vitro* studies demonstrate that FSH can enhance the survival and proliferation of these cancerous cells (Sanchez et al., 2016). Additionally, FSH, as a major regulator of estrogen, may contribute to the dysregulation of estrogen levels, further influencing the pathogenesis of breast cancer. The interactions between FSH and estrogen may explain the complexity of breast cancer treatment in postmenopausal patients (Yager and Davidson, 2006).

#### 7.1.3 Prostate cancer

Prostate cancer is the most common cancer among men and holds second highest cancer-related mortality in this population. Androgen plays a significant role in prostate cancer pathogenesis. Given this, androgen deprivation therapy, which blocks androgen signaling, is currently the standard treatment strategy for prostate cancer (Shafi et al., 2013; Desai et al., 2021; Zhang et al., 2022). The relationship between FSH and prostate cancer remains unclear due to a lack of clinical evidence. However, studies have detected FSHRs in several human prostate cancer cell lines, including PC-3, LNCaP, and C4-2. In these studies, administration of FSH has been shown to increase prostate-specific antigen levels and promote the proliferation of PC-3 cells (Dizeyi et al., 2019). Additionally, FSH signals to Sertoli cells to stimulate the production of androgen-binding proteins, which concentrate testosterone in the reproductive system (Oduwole et al., 2021). This effect of FSH suggests a potential role in amplifying androgen effects within the prostate gland, warranting further investigation into the impact of FSH on androgen levels in the prostate gland and its potential role in prostate cancer progression.

### 7.2 The underlying mechanisms

#### 7.2.1 Ovarian cancer

FSH primarily targets the reproductive tissues, including the ovarian surface epithelium. FSHRs are abundantly expressed on ovaries and exist in various isoforms that activate distinct downstream pathways involved in non-reproductive functions. These pathways support the growth, survival, metastasis, and apoptosis evasion of ovarian cancer cells (Zheng et al., 1996).

##### 7.2.1.1 ERK activation and calcium influx

FSH stimulates ovarian cancer cell growth by interacting with the FSH receptor variant FSHR3. Unlike FSHR1, which primarily operates through cAMP signaling in reproductive cells, FSHR3 activates the MAPK/ERK pathway and increases calcium influx independent of cAMP (Li et al., 2007; Sullivan et al., 2013). In the ovarian cancer cell line ID8, studies have demonstrated that FSHR3 activation is associated with the proliferation of ovarian surface epithelial cells through ERK phosphorylation (Zheng et al., 1996). Inhibiting this pathway with the MEK inhibitor PD98059 reduces both ERK activation and cell proliferation, highlighting the significance of MAPK/ERK signaling in FSH-driven tumor growth (Zheng et al., 1996). Additionally, ERK1/2 activation via FSHR1 is implicated in promoting epithelial-mesenchymal transition in ovarian cancer cells, with epithelial growth factor playing a key role (Vergara et al., 2010).

##### 7.2.1.2 Octamer-binding transcription factor 4 (OCT4) overexpression

OCT4 is a key regulator of stem cell pluripotency and self-renewal. Studies have shown that the dysregulation of OCT4 has been highly associated with the pathogenesis and advancement of various cancers. Overexpression of OCT4 has been observed in multiple cancers, including lung, prostate, liver, and cervical cancers, as well as ovarian cancer (Liu et al., 2011; Iki and Pour, 2006; Wang et al., 2010). In ovarian cancer, nuclear OCT4 overexpression has been reported in ovarian cells, further suggesting OCT4 involvement in ovarian cancer progression (Zhang et al., 2013).

In ovarian cancer, FSH upregulates OCT4 expression via the ERK1/2 signaling pathway, contributing to tumor progression (Liu et al., 2015). Elevated OCT4 promotes ovarian cancer cell proliferation and growth by upregulating bone morphogenetic protein 4 (BMP4) and interacting with Lin28 (Ma et al., 2013). Additionally, OCT4 overexpression in cancer stem cells increases the expression of Notch, SRY-Box transcription factor 2 (Sox2) and Nanog, which are crucial for stem cell proliferation and differentiation (Zhang et al., 2013).

OCT4 is also critical for promoting epithelial-mesenchymal transition in ovarian cancer cells. FSH-induced OCT4 overexpression increases Snail levels, leading to the downregulation of E-cadherin and the upregulation of N-cadherin, both of which are markers of epithelial-mesenchymal transition (Liu et al., 2015). Furthermore, OCT4 is involved in inhibiting apoptosis in ovarian cancer stem cells, linked to the activation of the AKT-survivin pathway (Hu et al., 2008). Increased OCT4 expression increases AKT and survivin levels, highlighting the importance of the OCT4-AKT-survivin axis in FSH-mediated apoptosis resistance (Zhang et al., 2013).

##### 7.2.1.3 Snail activation

In addition to FSH-induced OCT4 upregulation that increases Snail expression, another pathway involving FSH binding to FSHR1 has been identified. This binding activates CREB via the Gαs-cAMP-PKA signaling pathway. PKA moves to the nucleus, where it phosphorylates CREB, allowing it to act as a transcription factor (Zhang et al., 2021). Phosphorylated CREB then boosts human Alk B homolog 5 (ALKBH5) expression, a demethylase that removes m6A modifications from Snail mRNA. This epigenetic change extends the half-life of Snail mRNA, enhancing its role in promoting epithelial-mesenchymal transition by downregulating E-cadherin and upregulating N-cadherin, aiding cancer metastasis (Wang et al., 2023; Xu et al., 2024; Aborisade et al., 2022).

##### 7.2.1.4 Survivin upregulation, PDCD6 and DR5 downregulation

**FIGURE 3:**
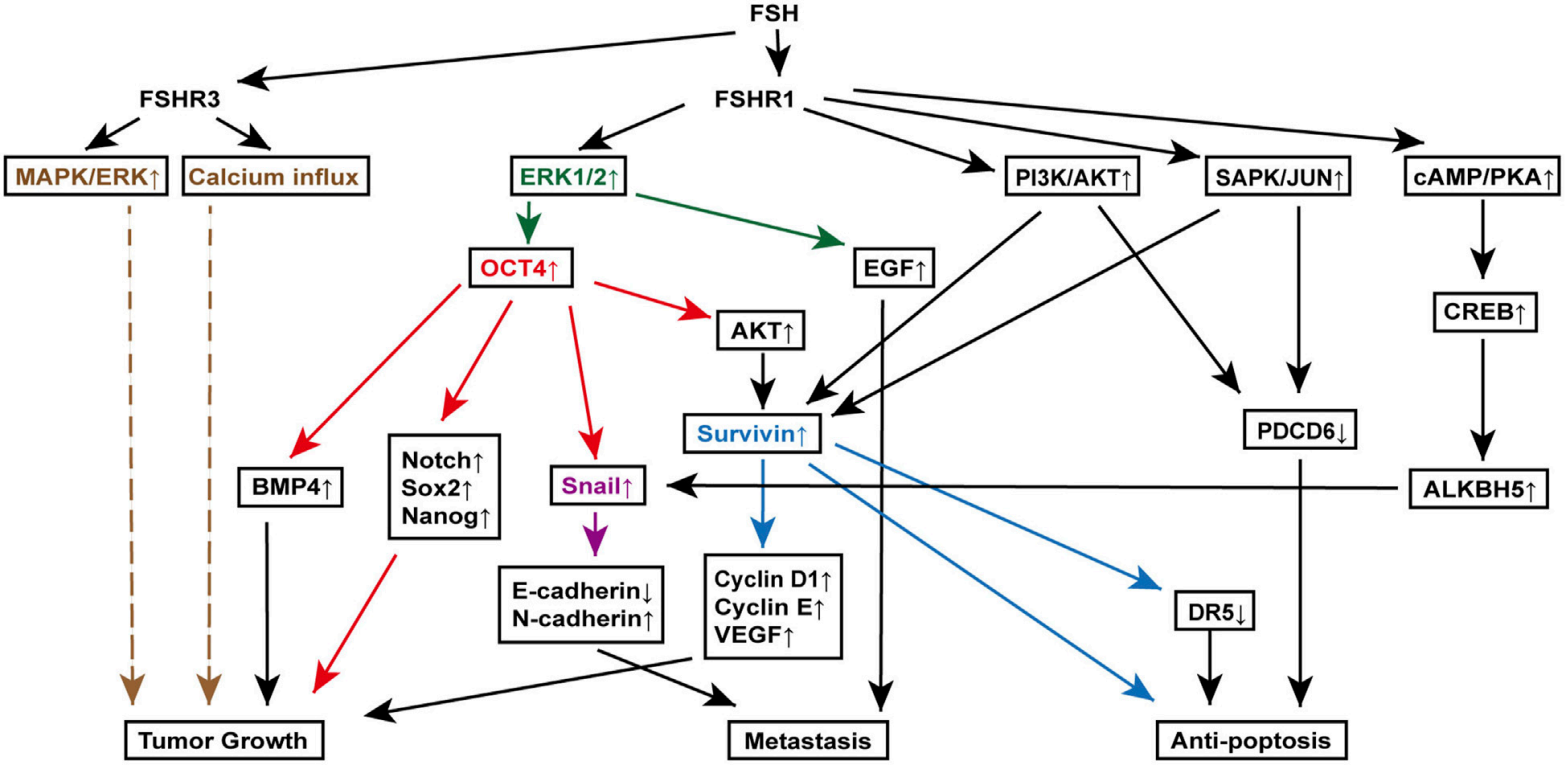
FSH signaling pathways involved in various mechanisms of ovarian cancer pathogenesis. Key molecules, extracellular signal-regulated kinase (ERK) (orange), octamer-binding transcription factor 4 (OCT4) (red), Snail (purple), and Survivin (blue), are highlighted to clearly delineate their downstream signaling roles in promoting tumor growth, metastasis, and anti-apoptosis. This figure illustrates the complex interactions between FSH receptors (FSHR1 and FSHR3) and various downstream effectors, leading to distinct cellular responses that contribute to the progression of ovarian cancer. Specifically, it showcases the roles of the mitogen-activated protein kinase (MAPK)/ERK pathway, OCT4 overexpression in epithelial-mesenchymal transition and cancer stem cell maintenance, Snail activation in metastasis, and Survivin-mediated resistance to apoptosis.

Survivin, an inhibitor of apoptosis, is upregulated in various cancers, including ovarian cancer, and plays a critical role in cell proliferation (Kondapuram et al., 2023). Yan et al. discovered that FSH binding to FSHR1 on ovarian cancer cells increases survivin expression through the PI3K/AKT pathway. This rise in survivin boosts VEGF expression, promoting ovarian cancer cell proliferation (Huang et al., 2008). Additionally, FSH treatment raises cyclin D1 and cyclin E levels while reducing programmed cell death gene 6 (PDCD6) and death receptor 5 (DR5) levels (Chu et al., 2021; Montalto and De Amicis, 2020). Further investigations using RNA interference to knock down survivin have shown that the changes in cyclin D1, cyclin E, and DR5 are survivin-dependent, while PDCD6 regulation appears independent of survivin. Moreover, FSH-induced survivin upregulation and PDCD6 downregulation are indicated to be downstream to the PI3K/AKT and stress-activated protein kinase/Jun N-terminal kinase (SAPK/JNK) signaling pathway, which are activated by the FSH binding to FSHR1 (Huang et al., 2011) (Figure 3).

#### 7.2.2 Breast cancer

FSH contributes to breast cancer growth and survival by promoting cell migration and invasion. It achieves this by modulating the actin cytoskeleton through the upregulation of Moesin and focal adhesion kinase (FAK) via ROCK-2 activation, which is downstream of Gα13 signaling and a Gαi and c-Src-dependent pathway triggered by FSH binding to FSHR. These pathways reorganize the cytoskeleton, enhancing cancer cell motility (Sanchez et al., 2016).

Additionally, FSH increases chemoresistance in breast cancer cells to drugs like doxorubicin and cyclophosphamide. This resistance is driven by the activation of hypoxia-inducible factor-1α (HIF-1α), a key regulatory protein that promotes the expression of genes in angiogenesis and glycolysis, thereby boosting the resilience of cancer cells to hypoxia and chemotherapy. FSH activates the PI3K/AKT pathway through FSHR interaction with Gαq/11, subsequently resulting in the inhibition of prolyl hydroxylases and an increase in intracellular HIF-1α levels (Bergandi et al., 2019).

### 7.3 Treatment strategies

Currently, there is no established clinical therapy targeting FSH singling for cancer treatment. However, preclinical studies suggest that blocking FSH-FSHR signaling with either FSH antagonists or FSHR antibodies is effective and safe to eliminate ovarian cancer in animal models (Blaakaer et al., 1995; Bordoloi et al., 2022; Perales-Puchalt et al., 2017).

Given the numerous signaling pathways involved in FSH-induced ovarian cancer progression and survival, targeted therapies against OCT4, ERK, Snail, and PDCD6 are also under investigation. Blocking these pathways has shown promise in inhibiting tumor growth, metastasis, and apoptosis escape in ovarian cancer cells (Liang et al., 2023; Ashraf and Kumar, 2022; Hojo et al., 2018; Yuan et al., 2017). For example, using short hairpin RNA to knockdown Snail in ovarian cancer cell lines largely reduced the proliferation rate of cancer cells, as well as reversed their chemoresistance to cisplatin, providing a better treatment effect and prognosis with chemotherapies (Hojo et al., 2018). Another therapeutic strategy using miR-124 also showed promising effect in the treatment of ovarian cancer. And the underlying mechanism is that miR-124 suppresses the expression of PDCD6, thus inhibiting the invasion and apoptosis evasion of cancer cells (Yuan et al., 2017). Although the precise targeting of cancer cells remains challenging, advancements in nanotechnology, histological techniques, and a deeper understanding of cancer biology may enable these therapeutic strategies to be translated into clinical practice, offering new hope to patients with FSH-related ovarian cancer or even other cancers with these pathogenic pathways.

In the context of breast and prostate cancer, hormonal therapies that reduce estrogen and androgen levels, respectively, have been well established and have proven effective in limiting cancer progression. For breast cancer, methods downregulating estrogen level, such as estrogen receptor modulators, aromatase inhibitors and ovarian suppressors, are widely employed. In prostate cancer, androgen deprivation therapy is the standard treatment (Nabid et al., 2018; Elsamany et al., 2022). However, adverse effects associated with the upregulated FSH, arising from negative feedback mechanisms due to reduced estrogen or androgen levels, can significantly worsen the physiological conditions of patients. These effects may compromise the overall outcomes of primary cancer treatments and contribute to the development of complications, including osteoporosis, sexual dysfunction, infertility, and cardiovascular disease (Maughan et al., 2010; Nguyen et al., 2015). As a result, the exploration of adjuvant therapies that specifically target FSH regulation, in order to maintain its physiological balance, represents a potential area for future research.

However, the direct role of FSH in breast cancer and prostate cancer remains poorly understood, and no FSH-targeted therapies have been developed to date. Given the known interactions between FSH and estrogen or androgen pathways, future research may focus on targeting FSH to indirectly suppress tumor initiation or progression.

## 8 Conclusion and discussion

The non-reproductive effects of FSH and their role in the pathogenesis of various diseases have been increasingly elucidated, revealing complex interactions between FSH signaling and a multitude of biological processes in various tissues and organs. The signaling pathways activated by FSH binding to its receptor, FSHR, have been well-characterized in many contexts. However, due to the complexity of FSHRs and their underlying signaling pathways, some downstream pathways remain incompletely understood. For instance, the downstream pathways of FSHR3 in ovarian cancer is not fully established even with a clear result of enhanced cell proliferation. Future advancements in the understanding of FSH signaling and intracellular molecular interactions are expected to further clarify these mechanisms, potentially leading to more precise and accurate models of FSH-related signaling cascades.

Although we have illustrated some of the underlying signaling pathways downstream FSH-FSHR signaling in various non-reproductive cells, the complexity of FSH reactions and signaling in the whole human body remains poorly understood. Several studies indicate that elevated FSH levels do not significantly impact bone, adipose tissue, cholesterol metabolism, or other non-reproductive functions (Liu et al., 2017; Wu et al., 2021; Elenkov et al., 2023). However, these studies primarily focus on changes in FSH levels without considering additional variables such as age, physiological conditions, and the levels of other hormones. Such gaps in endocrinological research highlight the necessity for comprehensive investigations into the effects of FSH within the entire organism.

Another critical area that underscores the importance of understanding FSH in the broader context is its role in male non-reproductive diseases, which remains inadequately explored. Although some studies suggest that elevated FSH may exacerbate several conditions, including cardiovascular disease, type 2 diabetes mellitus, and prostate cancer, the underlying mechanisms are often linked to the amplification of androgen effects resulting from FSH-androgen interactions (Wittert et al., 2021; Oduwole et al., 2021; Duivenvoorden et al., 2024; Shafi et al., 2013).

Additionally, while FSH influences various physiological processes that may, in turn, affect its own function, external factors can also modulate FSH levels. For instance, we previously noted that FSH may promote fat accumulation; however, a 2007 clinical trial involving Asian women found that lower FSH levels were associated with higher body mass index in postmenopausal women (Ausmanas et al., 2007). This condition may be attributed to excessive adipose tissue producing estrogen via aromatization, which exerts negative feedback on FSH production and secretion (De et al., 2006). Furthermore, estrogen from ovarian sources also complicates the interpretation of FSH functions, as FSH and estrogen levels are often interrelated under physiological conditions. Although many studies have demonstrated that FSH can perform non-reproductive functions independent of estrogen, the influence of estrogen on overall health cannot be overlooked. As research continues to delineate the effects of FSH on various non-reproductive tissues or cells, we might acquire a more comprehensive understanding of the FSH role in overall health.

Eventually, various treatments targeting FSH signaling, including FSH antibodies, FSHR blockade, and hormone replacement therapies, have been extensively studied across a range of diseases, with some now advancing to clinical trials. In conditions strongly associated with elevated FSH, such as osteoporosis, obesity, and Alzheimer’s disease, FSH-targeted therapies have shown significant promise. However, in diseases where FSH elevation is not a primary driver of pathogenesis, such as cardiovascular disease and certain cancers, FSH-suppressing treatments have not demonstrated clear therapeutic efficacy. This observation may suggest that FSH plays a less significant role in the development of these conditions. Nonetheless, FSH modulation may still hold potential as an adjunctive therapy, enhancing the efficacy of existing treatments and contributing to better clinical outcomes. This approach underscores the importance of precision medicine in the ongoing development of targeted therapies. To summarize, treatment strategies targeting FSH-FSHR signaling hold strong potential in both disease prevention and therapeutic interventions.

## Glossary

Aβ: amyloid-β
AEP: asparagine endopeptidase
AKT: protein kinase B
ALKBH5: human Alk B homolog 5
APP: amyloid precursor protein
BAT: brown adipose tissue
BMP4: bone morphogenetic protein 4
cAMP: cyclic adenosine monophosphate
C/EBPα: CCAAT/Enhancer binding protein alpha
CIDEA: cell death-inducing DFFA-like effector A
CREB: cAMP response element-binding receptors
DR5: death receptor 5
ERK: extracellular signal-regulated kinase
FAK: focal adhesion kinase
Fas: fatty acid synthase
FSH: follicle-stimulating hormone
FSHR: follicle-stimulating hormone receptor
GnRH: gonadotropin-releasing hormone
HMG-CoA reductase: 3-hydroxy-3-methyl glutaryl coenzyme A reductase
ICAM-1: intracellular adhesion molecule 1
IkBa: inhibitor of kappa B alpha
JNK: Jun N-terminal kinase
LDL-C: low-density lipoprotein cholesterol
LH: luteinizing hormone
Lpl: lipoprotein lipase
MAPK: mitogen-activated protein kinase
OCT4: octamer-binding transcription factor 4
PDCD6: programmed cell death gene 6
PI3K: phosphoinositide 3-kinase
PKA: protein kinase A
PPARγ: peroxisome proliferator-activated receptor gamma
SAPK: stress-activated protein kinase
Sox2: SRY-Box transcription factor 2
TC: total cholesterol
UCP1: uncoupling protein-1
VCAM-1: vascular cell adhesion molecule 1
VEGFA: vascular endothelial growth factor A
